# Detection of the Rhoptry Neck Protein Complex in *Plasmodium* Sporozoites and Its Contribution to Sporozoite Invasion of Salivary Glands

**DOI:** 10.1128/mSphere.00325-20

**Published:** 2020-08-19

**Authors:** Mamoru Nozaki, Minami Baba, Mayumi Tachibana, Naohito Tokunaga, Motomi Torii, Tomoko Ishino

**Affiliations:** a Division of Molecular Parasitology, Proteo-Science Center, Ehime University, Matsuyama, Ehime, Japan; b Division of Malaria Research, Proteo-Science Center, Ehime University, Matsuyama, Ehime, Japan; Johns Hopkins Bloomberg School of Public Health

**Keywords:** sporozoite, rhoptry, RON complex, salivary gland, *Plasmodium*, invasion

## Abstract

In the *Plasmodium* life cycle, two infectious stages of parasites, merozoites and sporozoites, share rhoptry and microneme apical structures. A crucial step during merozoite invasion of erythrocytes is the discharge to the host cell membrane of some rhoptry neck proteins as a complex, followed by the formation of a moving junction involving the parasite-secreted protein AMA1 on the parasite membrane. Components of the merozoite rhoptry neck protein complex are also expressed in sporozoites, namely, RON2, RON4, and RON5, suggesting that invasion mechanism elements might be conserved between these infective stages. Recently, we demonstrated that RON2 is required for sporozoite invasion of mosquito salivary gland cells and mammalian hepatocytes, using a sporozoite stage-specific gene knockdown strategy in the rodent malaria parasite model, Plasmodium berghei. Here, we use a coimmunoprecipitation assay and oocyst-derived sporozoite extracts to demonstrate that RON2, RON4, and RON5 also form a complex in sporozoites. The sporozoite stage-specific gene knockdown strategy revealed that both RON4 and RON5 have crucial roles during sporozoite invasion of salivary glands, including a significantly reduced attachment ability required for the onset of gliding. Further analyses indicated that RON2 and RON4 reciprocally affect trafficking to rhoptries in developing sporozoites, while RON5 is independently transported. These findings indicate that the interaction between RON2 and RON4 contributes to their stability and trafficking to rhoptries, in addition to involvement in sporozoite attachment.

**IMPORTANCE** Sporozoites are the motile infectious stage that mediates malaria parasite transmission from mosquitoes to the mammalian host. This study addresses the question whether the rhoptry neck protein complex forms and functions in sporozoites, in addition to its role in merozoites. By applying coimmunoprecipitation and sporozoite stage-specific gene knockdown assays, it was demonstrated that RON2, RON4, and RON5 form a complex and are involved in sporozoite invasion of salivary glands via their attachment ability. These findings shed light on the conserved invasion mechanisms among apicomplexan infective stages. In addition, the sporozoite stage-specific gene knockdown system has revealed for the first time in *Plasmodium* that the RON2 and RON4 interaction reciprocally affects their stability and trafficking to rhoptries. Our study raises the possibility that the RON complex functions during sporozoite maturation as well as migration toward and invasion of target cells.

## Abstract (precis)

Sporozoites are the motile infectious stage that mediates malaria parasite transmission from mosquitoes to the mammalian host. This study addresses the question whether the rhoptry neck protein complex forms and functions in sporozoites, in addition to its role in merozoites. By applying coimmunoprecipitation and sporozoite stage-specific gene knockdown assays, it was demonstrated that RON2, RON4, and RON5 form a complex and are involved in sporozoite invasion of salivary glands via their attachment ability. These findings shed light on the conserved invasion mechanisms among apicomplexan infective stages. In addition, the sporozoite stage-specific gene knockdown system has revealed for the first time in *Plasmodium* that the RON2 and RON4 interaction reciprocally affects their stability and trafficking to rhoptries. Our study raises the possibility that the RON complex functions during sporozoite maturation as well as migration toward and invasion of target cells.

## INTRODUCTION

Infective forms of apicomplexan parasites, including *Plasmodium* merozoites, sporozoites, and *Toxoplasma* tachyzoites, share apical secretory organelles named micronemes and rhoptries. The proper discharge of rhoptry proteins is essential for host cell invasion by *Toxoplasma* tachyzoites and *Plasmodium* merozoites, which was demonstrated by knockdown of a lipid binding protein, rhoptry apical surface protein 2, required for rhoptry protein secretion (1). Some proteins localized to the rhoptry neck region are inserted into the host cellular membrane as a complex prior to invasion and interact with a protein on the parasite membrane, apical membrane antigen 1 (AMA1), to form a moving junction between the parasite and target cells (2–5). The importance of the interaction between rhoptry neck protein 2 (RON2) and AMA1 for merozoite invasion of erythrocytes has been shown using inhibitory antibodies and peptides (6–9). The inability to target gene disruption of *ron2*, *ron4*, or *ron5* within the *Plasmodium* genome supports a model in which components of the RON complex are crucial for merozoite invasion (10, 11). In *Toxoplasma* tachyzoites, RON5 conditional knockdown revealed that the protein is required for parasite invasion, possibly via stabilizing RON2 and transportation of RON4 (12). Consistent with reports that the RON2-AMA1 interaction is an important step for parasite invasion, AMA1 knockdown resulted in a failure of Plasmodium falciparum merozoite invasion (13). However, the contribution of AMA1 in the *Plasmodium* life cycle has been questioned as *ama1* gene-disrupted parasites were generated in Plasmodium berghei, a rodent malaria parasite model strain (14). AMA1 functional complementation has not been reported in *Plasmodium*, while its redundancy has been demonstrated in *Toxoplasma* (15).

Sporozoites are another infectious stage in the *Plasmodium* life cycle and are transmitted from mosquito vectors to mammalian hosts. Sporozoites, formed inside oocysts on the mosquito midgut wall, invade mosquito salivary glands to be later released in the mammalian skin during mosquito probing and then migrate to the liver via blood vessels to infect hepatocytes. It has been demonstrated that most rhoptry proteins detected in merozoites also localize to rhoptries in sporozoites, except for the high-molecular-mass rhoptry protein (RhopH) complex components (10, 16, 17). The sporozoite-stage-specific gene silencing system in P. berghei has revealed that RON2 is required for sporozoite invasion of salivary glands of mosquitoes as well as infection of the mouse liver, possibly via participation in sporozoite attachment ability and motility (18). Sporozoite motility is involved in salivary gland invasion and migration toward the liver after injection in the mammalian skin. It has been demonstrated that sporozoite motility is mediated by sporozoite-stage-specific secretory proteins stored in micronemes, such as thrombospondin-related adhesive protein (TRAP) (19, 20), TRAP-related protein/upregulated in oocyst sporozoite 3 (TREP/S6/UOS3) (21–23), and sporozoite invasion association protein-1 (SIAP-1) (24). The sporozoite surface protein LIMP was demonstrated to be involved in both attachment and motility (25). RON2 is the first rhoptry protein demonstrated to be involved in sporozoite attachment and motility. In addition, RON4 has been shown to be secreted and required for sporozoite infection of hepatocytes *in vitro* (26, 27). For further elucidation of the mechanisms mediating sporozoite invasion of mosquito salivary gland cells and mammalian hepatocytes, we aimed to address the question whether the RON complex is formed in sporozoites and to investigate its function during sporozoite invasion.

In this study, we use the P. berghei model to demonstrate by coimmunoprecipitation that a RON complex composed of RON2, RON4, and RON5 is also formed in oocyst-derived sporozoites. Repression of RON4 or RON5 expression in sporozoites using promoter swapping demonstrates that RON4 and RON5 are crucial for sporozoite invasion of salivary glands, similar to RON2. Detailed analyses revealed that RON2 and RON4 reciprocally affect stability and/or correct localization to rhoptries, whereas RON5 is not affected by their knockdown. This study indicates that RON2 and RON4 interaction is required for their trafficking to rhoptries, and all components of the RON complex are crucial for sporozoite invasion of salivary glands, via mediating attachment ability and motility.

## RESULTS

### Rhoptry neck protein complex components are expressed in sporozoites.

The RON complex components formed at the moving junction of *Plasmodium* merozoites, RON2, RON4, and RON5, also localize to rhoptries in sporozoites (16). Specific antibodies against RON4 and RON5 were prepared to characterize RON4 and RON5 during P. berghei sporozoite maturation in mosquitoes and to determine whether the RON complex forms in sporozoites. Recombinant proteins corresponding to the C-terminal regions of RON4 (amino acids 565 to 786, RON4C) and RON5 (amino acids 861 to 1148, RON5C) were expressed with glutathione *S*-transferase (GST) tags at their N terminus using a wheat germ cell-free protein production system and used for rabbit immunization. The reactivity and specificity of antibodies against RON4 and RON5 were examined by Western blotting using schizont extracts from transgenic parasites expressing RON4 or RON5 tagged with c-Myc at their C terminus (16). Anti-RON4C antibodies specifically recognize full-length and processed forms of RON4, at approximately 110 and 70 kDa, respectively, as reported previously (16) (see Fig. S1A in the supplemental material). Anti-RON5C antibodies detect predicted full-length RON5 at approximately 130 kDa, with a few minor bands which were not recognized by anti-c-Myc antibodies and therefore are presumably nonspecific or degradation products (Fig. S1B).

10.1128/mSphere.00325-20.1 FIG S1 Reactivity and specificity of specific antibodies against RON4 and RON5. Lysates of 2 × 10^5^ schizonts of RON4-c-Myc or RON5-c-Myc were separated by SDS-PAGE on 5 to 20% gradient acrylamide gels and transferred to membranes. The membranes were incubated with anti-c-Myc antibodies (1:500), anti-RON4-C antibodies (0.4 μg/ml), or anti-RON5-C antibodies (3.4 μg/ml), followed by secondary antibodies conjugated to horseradish peroxidase. (A) Specific signals corresponding to RON4 full-length and processed forms are indicated by open and closed arrowheads, respectively. Anti-RON4 antibodies specifically recognized RON4 tagged by c-Myc at the C terminus. (B) RON5 tagged by c-Myc was detected by anti-RON5-C antibodies as the full-length form, indicated by an open arrowhead. Download FIG S1, TIF file, 2.6 MB. Copyright © 2020 Nozaki et al. 2020 Nozaki et al. This content is distributed under the terms of the Creative Commons Attribution 4.0 International license.

**FIG 1:**
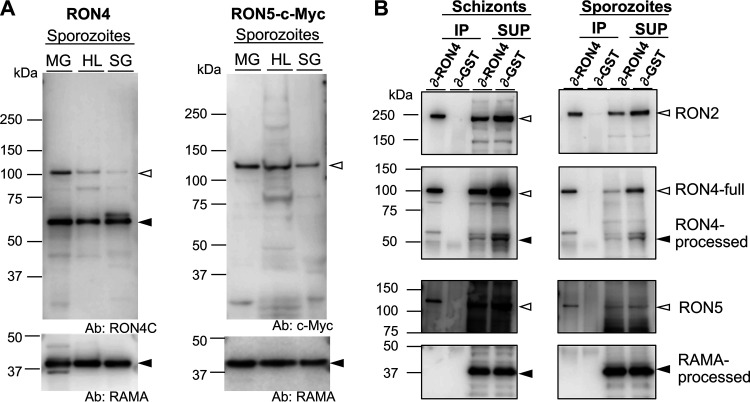
RON2, RON4, and RON5 form a complex in oocyst-derived sporozoites as well as in merozoites. (A) Expression profiling of representative rhoptry proteins during sporozoite maturation in mosquito bodies. Sporozoites were collected from midguts (MG), hemolymph (HL), and salivary glands (SG) of *Pb*WT-GFP- or RON5-c-Myc-infected mosquitoes at 22 to 24 days postfeeding. Protein homogenates from 50,000 or 75,000 sporozoites, for RON4 or RON5 assays, respectively, were separated by SDS-PAGE and transferred to membranes, prior to detection by specific antibodies against RON4, c-Myc, and RAMA. Predicted full-length RON4 (left panel) and RON5 (right panel) proteins are indicated by open arrowheads. The processed forms of RON4 (left panel) and RAMA (lower panels) are indicated by closed arrowheads. (B) Lysates of schizonts (left panels) and oocyst-derived sporozoites (right panels) were immunoprecipitated with anti-RON4 or anti-GST antibodies. RON2, RON4, and RON5 were detected in antibody-bound proteins (immunoprecipitation [IP]) and nonbound proteins (supernatant [SUP]) by Western blotting. Specific protein bands corresponding to the full-length and processed forms are indicated by open and closed arrowheads, respectively. The mobility of the RON4 processed form in the IP sample was slightly lower, possibly due to inhibition by antibodies for immunoprecipitation (about 50 kDa). RON2 and RON5 were coimmunoprecipitated with RON4 in both merozoites and sporozoites.

To examine RON4 and RON5 protein amounts and processing patterns during sporozoite maturation in mosquitoes, Western blotting was performed using sporozoites collected from midguts, hemolymph, and salivary glands of P. berghei wild-type–green fluorescent protein (*Pb*WT-GFP) or RON5-c-Myc parasite-infected mosquitoes (Fig. 1A). RON4 was detected as two bands corresponding to full-length and processed forms in oocyst-derived sporozoites, and the full-length signal intensity decreased as sporozoites matured. RON5-c-Myc-expressing transgenic sporozoites and anti-c-Myc antibodies were used to detect RON5 protein amounts specifically during sporozoite maturation in mosquitoes. RON5-c-Myc was detected at its predicted size through sporozoite maturation, although its amount slightly decreases after sporozoite invasion of salivary glands. For a loading control, the processed form of rhoptry-associated membrane antigen (RAMA) was detected by incubating the same membrane with anti-RAMA antibodies (Fig. 1A, lower panels).

### RON2, RON4, and RON5 form a complex in oocyst-derived sporozoites.

To investigate whether the RON complex is formed in P. berghei sporozoites, an immunoprecipitation assay was performed using anti-RON4C antibodies. Sporozoites collected from mosquito midguts, and a schizont-enriched sample as a control, were treated in 1% CHAPS {3-[(3-cholamidylpropyl)-dimethylammonio]-1-propanesulfonate} and sonicated to solubilize the rhoptry proteins. The protein lysates were then incubated with anti-RON4C antibodies, or anti-GST antibodies as a negative control, to precipitate antibody-bound proteins. Coprecipitated proteins (IP) were analyzed by Western blotting and compared with nonbound proteins (SUP). RON2 and RON5 proteins, together with RON4, were detected in anti-RON4C antibody-precipitated samples from both schizonts and oocyst-derived sporozoites (Fig. 1B). RON complex formation in sporozoites was further confirmed by coimmunoprecipitation using anti-RON2N antibodies and extracts from RON5-c-Myc-expressing oocyst-derived sporozoites (Fig. S2A). Taken together, RON2, RON4, and RON5 are demonstrated to also form a complex in oocyst-derived sporozoites. It is noteworthy that the ratio of the RON5 to RON2 amounts in precipitates was smaller from sporozoite lysates than schizont-enriched protein lysates. We next attempted to address the question whether the RON complex in sporozoites could interact with AMA1, a known RON complex-interacting merozoite micronemal protein. The results from two independent experiments, shown in Fig. S2B, suggest that AMA1 was present in anti-RON4 antibody immunoprecipitates; however, the signal intensity was far weaker from sporozoite than from schizont precipitants. Therefore, it was not clear whether this interaction is functionally important for sporozoite invasion of target cells.

10.1128/mSphere.00325-20.2 FIG S2 Confirmation of rhoptry neck protein complex formation using anti-RON2 antibodies for coimmunoprecipitation. (A) Lysates of schizonts (left) and oocyst-derived sporozoites (right) of RON5-c-Myc-expressing parasites were immunoprecipitated with anti-RON2 or anti-GST antibodies. RON2, RON4, and RON5-c-Myc were detected in lysates and following immunoprecipitation (IP) by Western blotting using anti-RON2, anti-RON4, and anti-c-Myc antibodies. Specific protein bands corresponding to the full-length (RON2, RON4, and RON5-c-Myc) and processed (RON4) form are indicated by open and closed arrowheads, respectively. RON4 and RON5 were coimmunoprecipitated with RON2 in both merozoites and sporozoites. (B) Detection of AMA1 in immunoprecipitations by anti-RON4 antibody from two independent experiments. Schizont or sporozoite lysates indicate the starting material for immunoprecipitation (IP). As AMA1 expression is not sufficiently abundant in oocyst-derived sporozoites, the number of sporozoites loaded per lane was four times that of schizonts. In schizont lysates AMA1 was detected repeatedly as a clear band at about 60 kDa (indicated by open arrowheads). From oocyst-derived sporozoites, very faint bands corresponding to AMA1 could be detected in RON4 antibody precipitations when the filter was exposed longer (lower panel). Download FIG S2, TIF file, 2.4 MB. Copyright © 2020 Nozaki et al. 2020 Nozaki et al. This content is distributed under the terms of the Creative Commons Attribution 4.0 International license.

### Generation of sporozoite-stage-specific *ron4* or *ron5* knockdown transgenic parasites.

**FIG 2:**
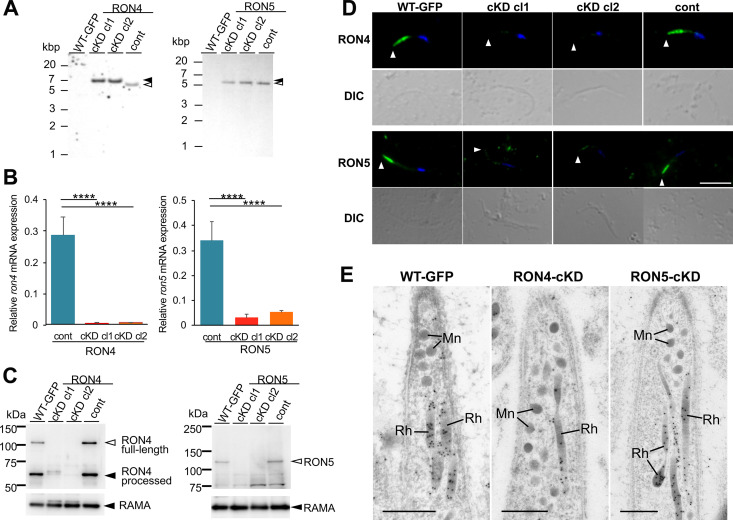
Generation of sporozoite-stage-specific *ron4* or *ron5* knockdown transgenic parasites. (A) Genomic Southern blot analyses using genomic DNA extracted from *Pb*WT-GFP and transgenic parasites. Genomic DNA was digested with BamHI and EcoRV, separated by size via agarose gel electrophoresis, and transferred to membranes. The signals were obtained by hybridization of the DNA probe within the hDHFR cassette at expected sizes indicated by closed and open arrowheads for conditional knockdown and control parasites, respectively. The specific bands at the expected size (6.4, 5.9, 5.7, and 5.5 kbp for RON4-cKD, RON4-cont, RON5-cKD, and RON5-cont, respectively) (see Fig. S3A and B) demonstrate that DNA insertion occurred at the correct locus to replace the original promoter regions. The DNA size marker is shown on the left of each image. (B) Relative amounts of *ron4* and *ron5* mRNA in oocyst-derived sporozoites. Total RNA was extracted from mosquito midguts collected at days 14 to 15 postfeeding that were infected with RON4-cont, RON4-cKD cl1, RON4-cKD cl2, RON5-cont, RON5-cKD cl1, and RON5-cKD cl2. The values were normalized by the expression of *pbhsp70* mRNA in each sample. Experiments were repeated at least four times with independently prepared sets of samples (see Fig. S3C), and the means with standard deviations are shown as bar graphs. Statistical differences were calculated by one-way ANOVA with Tukey’s multiple-comparison test (****, *P < *0.0001). (C) (Upper panels) Western blot analysis of RON4 and RON5 in oocyst-derived sporozoites. Protein homogenates of 100,000 sporozoites collected from mosquito midguts at days 24 to 26 after infection with *Pb*WT-GFP and transgenic parasites, indicated above the membrane, were separated by SDS-PAGE, and RON4 and RON5 proteins were detected using specific antibodies. Open and closed arrowheads indicate full-length RON4 (left panel) or RON5 (right panel) and processed RON4 (left panel), respectively. (Lower panels) Protein loading was confirmed by detecting RAMA indicated by closed arrowheads. The relative RON4 or RON5 band intensities, measured by ImageQuant TL, are shown in Fig. S3D. (D) Indirect immunofluorescence analysis of RON4 and RON5. Oocyst-derived sporozoites were stained with anti-RON4 or anti-RON5 antibodies (green). Nuclei are visualized with 4′,6-diamidino-2-indole (DAPI) in merged images (blue). Bright-field images are shown in the lower panels (DIC). In *Pb*WT-GFP and each of the control sporozoites, the specific signals corresponding to RON4 or RON5 were detected at the apical end; however, these signals were undetectable at the apical end (arrowheads) of RON4-cKD and RON5-cKD sporozoites. Bar, 5 μm. (E) Rhoptry formation and RAMA trafficking to rhoptries occur normally in RON4-cKD and RON5-cKD sporozoites. Immunoelectron microscopy analyses of RON4-cKD and RON5-cKD transgenic parasites. Midguts were dissected from mosquitoes infected with *Pb*WT-GFP, RON4-cKD, or RON5-cKD parasites on day 17 postfeeding and fixed. Ultrathin sections were stained with anti-RAMA antibodies followed by secondary antibodies conjugated to gold particles. Rhoptries and micronemes form at the apical-end region of transgenic sporozoites as well as *Pb*WT-GFP sporozoites. RAMA is localized to rhoptries correctly in transgenic sporozoites. Bars, 500 nm; Rh, rhoptry; Mn, microneme.

We reported previously that RON2 is involved in sporozoite invasion of salivary glands via its contribution to attachment ability (18). We next investigated whether its interaction partners, RON4 and RON5, have similar roles in sporozoites. Since RON4 and RON5 are possibly essential for intraerythrocytic-stage parasite development, their expression must be retained in the early schizont stage but otherwise repressed to elucidate their function in sporozoites. To generate sporozoite-stage-specific gene silencing transgenic parasites, the native promoter region for *pbron4* or *pbron5* in *Pb*WT-GFP genomic DNA was replaced by homologous recombination with a promoter region for *pb merozoite surface protein 9* (*msp9*, Fig. S3A and B), as it was demonstrated that swapping the *ron2* promoter with the *msp9* promoter reduced the RON2 amount in sporozoites by approximately 50-fold (18). Two independent clones were isolated for each transgenic parasite and designated RON4 conditional knockdown (RON4-cKD) and RON5 conditional knockdown (RON5-cKD). For the generation of control parasites, the native promoter regions were replaced with the *ron4* promoter (RON4-cont) and the *pb rhoptry associated protein 1* (*rap1*) promoter (RON5-cont), similar to the creation of RON2-control parasites (18). The DNA insertion at the correct genomic locus of transgenic parasites was confirmed by genomic Southern blotting using a DNA probe corresponding to the drug-resistant cassette region (Fig. 2A).

10.1128/mSphere.00325-20.3 FIG S3 Generation of RON4 or RON5 knockdown sporozoites. (A and B) Schematic representation of the *ron4* and *ron5* locus in wild-type and transgenic parasites. For the generation of knockdown parasites (RON4-cKD and RON5-cKD), the *ron4* and *ron5* promoter (*ron4p* and *ron5p*) regions were replaced with the *msp9* promoter region *(msp9p)* by homologous recombination. For the generation of control parasites (RON4-cont and RON5-cont), specific promoter regions were replaced by *ron4p* or *rap1* promoter (*rap1p*) regions, respectively. A human DHFR-expressing cassette (*hdhfr*) was inserted for drug selection of transgenic parasites. The regions used for homologous recombination are indicated as *ron4*-upstream (green bars), RON4-N (light green boxes), *ron5*-upstream (yellowish brown bars), and RON5-N (yellowish brown boxes). BamHI and EcoRV restriction sites and the probe region used for genomic Southern blot analysis are indicated (gray bars). (C) To demonstrate the relative *ron4* or *ron5* mRNA amounts in Fig. 2C, at least four independently prepared oocyst samples were used. Each relative value corresponding to *ron4* or *ron5* mRNA amounts, normalized by the *hsp70* mRNA amount, is presented as a table. (D) Quantitative analyses of RON4 and RON5 amounts normalized by RAMA amounts in RON4-cKD and RON5-cKD sporozoites. Relative band intensities of RON4 (integration of full-length and processed form), RON5, and RAMA from Western blotting data shown in Fig. 2C were measured by ImageQuant TL (GE Healthcare, United Kingdom) and shown as blue bars. Quantification data from differently prepared sporozoite samples are shown as orange bars. Download FIG S3, TIF file, 0.4 MB. Copyright © 2020 Nozaki et al. 2020 Nozaki et al. This content is distributed under the terms of the Creative Commons Attribution 4.0 International license.

To examine the reduction in mRNA transcripts in sporozoites, real-time reverse transcription (RT)-PCR analyses were performed using developing sporozoites in oocysts at days 14 to 15 after feeding on transgenic parasite-infected mice. The relative mRNA amounts of *ron4* in RON4-cKD clone 1 (cl1) and cl2 sporozoites were approximately 70- and 40-fold less than in RON4-cont sporozoites, respectively (Fig. 2B, left, and Fig. S3C). Similarly, the relative *ron5* mRNA amounts in RON5-cKD cl1 and cl2 sporozoites were approximately 11- and 7-fold less than in RON5-cont sporozoites, respectively (Fig. 2B, right, and Fig. S3C). In agreement with the transcript data, Western blots showed that clear bands corresponding to RON4 or RON5 proteins were absent in oocyst-derived RON4-cKD or RON5-cKD sporozoite extracts (Fig. 2C and Fig. S3D). Immunofluorescence analyses (IFAs) using specific antibodies confirmed that the typical apical-end localization of RON4 or RON5 in control sporozoites was diminished in RON4-cKD or RON5-cKD sporozoites (Fig. 2D). Morphological analysis by electron microscopy on oocyst-derived sporozoites of *ron4* or *ron5* knockdown transgenic parasites revealed that sporozoites are normally formed with a proper apical structure having micronemes and rhoptries, which are labeled with anti-RAMA antibodies (28) (Fig. 2E). These results indicate that the disappearance of the RON4 and RON5 apical-end signal by IFA was not due to rhoptry dysplasia or abnormal protein trafficking to rhoptries. Taken together, it was demonstrated that promoter swapping to an *msp9* promoter successfully represses RON4 or RON5 expression in oocyst-derived sporozoites, where their transcription predominantly occurs, and therefore these transgenic parasites were used for functional analyses of RON4 and RON5 in sporozoites.

### RON4 or RON5 knockdown affects sporozoite ability to invade mosquito salivary glands.

**FIG 3:**
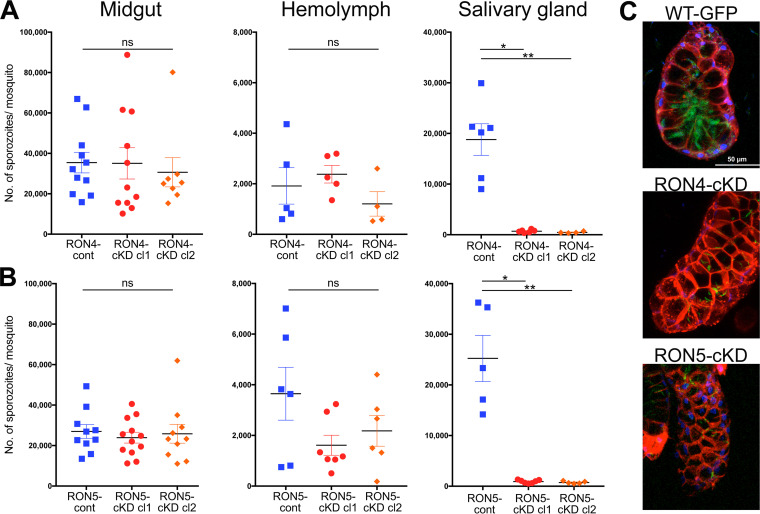
Repression of *ron4* or *ron5* expression decreases sporozoite invasion efficiency for mosquito salivary glands. Sporozoite numbers collected and counted from midguts, hemolymph, and salivary glands of transgenic parasite-infected mosquitoes at days 23 to 26 postfeeding. Each dot represents the average sporozoite numbers from 20 to 30 mosquitoes. The horizontal bars and error bars indicate the means and standard deviation from at least four independent experiments using independently prepared mosquito groups. (A) The numbers of sporozoites of RON4-cont, RON4-cKD cl1, and RON4-cKD cl2 are shown as dot plots. *P* values were calculated by using the Kruskal-Wallis test. No significant difference was observed in the number of sporozoites collected from midguts (*P* value: 0.7171, indicated as ns) or from hemolymph (*P* value: 0.4689, indicated as ns). In contrast, the number of sporozoites collected from salivary glands was significantly different (*P* value: 0.0001). The *P* value from *post hoc* analysis, the Dunn multiple-comparison test, is shown in the graph (**, *P* < 0.01; *, *P* < 0.05). (B) The numbers of sporozoites from RON5-cont, RON5-cKD cl1, and RON5-cKD cl2 are shown as dot plots. *P* values were calculated by using the Kruskal-Wallis test. No significant difference was observed in the number of sporozoites collected from midguts (*P* value: 0.8651, indicated as ns) or from hemolymph (*P* value: 0.3506, indicated as ns). In contrast, the number of sporozoites collected from salivary glands was significantly different (*P* value: 0.0008). The *P* value from *post hoc* analysis is shown in the graph (**, *P* < 0.01; *, *P* < 0.05). (C) Detection of RON4-cKD and RON5-cKD sporozoites inside salivary glands. Salivary glands of *Pb*WT-GFP-, RON4-cKD-, or RON5-cKD-infected mosquitoes were dissected at day 21 postfeeding and stained with FM4-64 (cellular membrane, red) and Hoechst stain (nuclei, blue) and then observed by confocal laser microscopy. Sporozoites were detected by the GFP signal (green). The number of RON4-cKD or RON5-cKD sporozoites residing in salivary glands is limited; however, their distribution in salivary glands is similar to that of *Pb*WT-GFP sporozoites. Merged images for 15- to 25-μm thickness are shown. Bar, 50 μm.

To investigate the roles of RON4 and RON5 in sporozoites on maturation and invasion of salivary glands, sporozoite numbers collected from midguts, hemolymph, and salivary glands of infected mosquitoes were determined for control and conditional knockdown parasites. Sporozoite numbers of RON4-cKD or RON5-cKD collected from midguts and hemolymph were not significantly different from those of control parasites (Fig. 3A and B, left and middle graphs), indicating that neither RON4 nor RON5 is critical for sporozoite formation, maturation inside oocysts, and release to the hemocoel. In contrast, the mean numbers of sporozoites residing in salivary glands were reduced 27- or 41-fold in cl1 and cl2 by *ron4* knockdown and 28- or 34-fold by *ron5* knockdown in the respective cl1 and cl2 clones (Fig. 3A and B, right graphs). To examine whether sporozoite maturation is delayed by RON4 and RON5 knockdown, infected mosquitoes were divided into three groups and salivary glands were dissected at days 21, 25, and 29 postfeeding. The numbers of sporozoites collected from salivary glands remained low through the time course for both RON4 and RON5 knockdown parasites (Fig. S4). The confocal images of salivary glands from mosquitoes infected by RON4-cKD or RON5-cKD parasites confirmed that the number of salivary gland-residing sporozoites is far smaller than for *Pb*WT-GFP (Fig. 3C). These results demonstrate that RON4 and RON5 have crucial roles required for sporozoite invasion of salivary glands, similarly to RON2 (18).

10.1128/mSphere.00325-20.4 FIG S4 Transition of the number of sporozoites residing in salivary glands. A group of infected mosquitoes was divided into three groups and dissected to collect salivary glands at days 21, 25, and 29 postfeeding. Each dot represents the average sporozoite numbers from 19 to 25 mosquitoes infected by *Pb*WT-GFP (blue diamonds), RON4-cont (blue triangles), RON4-cKD (red triangles), RON5-cont (blue circles), or RON5-cKD (pink circles). The number of RON4-cKD or RON5-cKD sporozoites collected from salivary glands remained low through the time course. Download FIG S4, TIF file, 0.1 MB. Copyright © 2020 Nozaki et al. 2020 Nozaki et al. This content is distributed under the terms of the Creative Commons Attribution 4.0 International license.

### RON4-cKD and RON5-cKD sporozoites demonstrate impaired adhesion ability.

**FIG 4:**
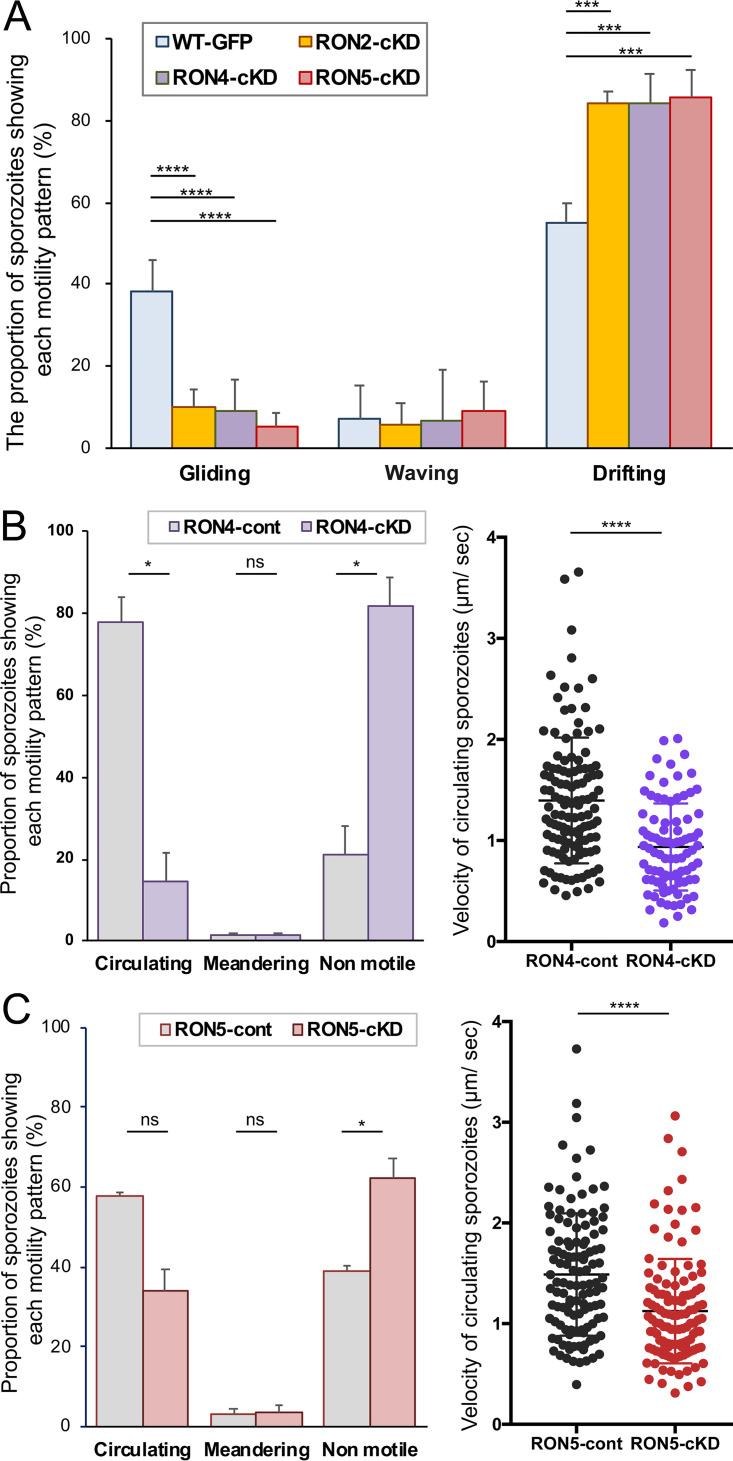
RON4 and RON5 are important for hemolymph sporozoite attachment. (A) The sporozoite motility patterns on glass slides for RON2-cKD, RON4-cKD, and RON5-cKD transgenic parasites were compared with that of *Pb*WT-GFP. Sporozoites collected from hemolymph of infected mosquitoes were activated by incubation in 10% FCS-containing medium, and their movement patterns were classified into three categories: gliding, waving, and drifting. At least 75 hemolymph sporozoites were observed for each parasite line, and the mean proportions showing each moving pattern from five independent experiments are shown as a bar graph with standard deviations. *P* values were calculated by the one-way ANOVA test with the Tukey multiple-comparison test (****, *P* < 0.0001; ***, *P* < 0.001). (B and C) The motile patterns of hemolymph sporozoites of RON4-cKD (B) and RON5-cKD (C) in Matrigel are shown (left graphs). Motile patterns were classified into three categories: circulating, meandering, and nonmotile, as described previously (18). The mean values from four independent experiments are shown as bar graphs with standard deviations. The statistical differences in the percentage of sporozoites with each moving pattern between control (RON4-cont and RON5-cont) and knockdown (RON4-cKD and RON5-cKD) were calculated by the Mann-Whitney *U* test (*, *P* < 0.05; ns, not significant). The velocity of circulating sporozoites (*n* > 88) in the Matrigel was calculated by Fiji software and plotted in the right graphs. The statistical differences in the velocity between control (RON4-cont and RON5-cont) and knockdown (RON4-cKD and RON5-cKD) were calculated by the Mann-Whitney *U* test (****, *P* < 0.0001).

To invade salivary glands, sporozoites need the ability to adhere, followed by gliding motility. These processes are mediated by the secretion of micronemal proteins, such as TRAP, TREP/S6/UOS3, SIAP-1, and MAEBL (19–24, 29, 30), together with a surface protein, LIMP (25). Recently, we demonstrated that RON2 is involved in sporozoite adhesion, which is required for the onset of gliding (18). Therefore, we assessed the *in vitro* motility and attachment ability of RON4-cKD and RON5-cKD sporozoites collected from hemolymph in comparison to that of RON2-cKD sporozoites. As a reference, 38% of *Pb*WT-GFP hemolymph sporozoites start gliding, while 55% remain floating without attachment to the glass slide, categorized as drifting (Fig. 4A). In contrast, approximately 85% of RON4-cKD and RON5-cKD hemolymph sporozoites drifted, similar to the phenotypes of RON2-cKD hemolymph sporozoites (18). These results indicate that RON4 and RON5 are required for hemolymph sporozoite attachment to the glass slide similarly to RON2. Accordingly, only 9% and 5% of RON4-cKD and RON5-cKD hemolymph sporozoites show gliding, which is 4- and 7-fold less than *Pb*WT-GFP gliding sporozoites, respectively, consistent with a report that sporozoites need to attach to the glass slide at both ends prior to the initiation of gliding (31, 32). We could not detect secreted RON4 or RON5 on the trail of *Pb*WT-GFP gliding sporozoites, possibly because the amount of secreted RON4 or RON5 is far less than those stored in rhoptries or because they are not aggregated with the trail (Fig. S5).

10.1128/mSphere.00325-20.5 FIG S5 Neither RON4 nor RON5 is deposited in trails after sporozoite gliding. Sporozoites collected from hemolymph were incubated with RPMI 1640 medium containing 10% FCS for 45 min at 37°C in an 8-well chamber slide. After fixation with 10% formalin, sporozoites were permeabilized with 0.1% Triton X-100. Trails were visualized by anti-circumsporozoite protein (CSP) antibodies (red), and nuclei were stained with DAPI (cyan) in merged images. RON4 (A) and RON5 (B) were detected inside sporozoites by anti-RON4C antibodies and anti-RON5C antibodies but not on trails (green). Bright-field images were shown at the right (DIC). Download FIG S5, TIF file, 1.8 MB. Copyright © 2020 Nozaki et al. 2020 Nozaki et al. This content is distributed under the terms of the Creative Commons Attribution 4.0 International license.

Next, hemolymph sporozoites were embedded in Matrigel, composed of laminin and type IV collagen and mimicking the environment of sporozoites inoculated into mammalian skin by mosquitoes, and their moving patterns were analyzed by the categories described by Volkmann et al. (33). When embedded in Matrigel, 78% of control hemolymph sporozoites move continuously through the matrix, whereas only 15% of RON4-cKD hemolymph sporozoites display a circular mode of motility (Fig. 4B). In contrast, the ratio of continuously circulating RON5-cKD hemolymph sporozoites was approximately 34% (Fig. 4C). The ratio of continuously migrating RON4-cKD and RON5-cKD hemolymph sporozoites is increased 1.7- and 6.8-fold by embedding in Matrigel, respectively, possibly due to a Matrigel-mediated bypassing of the requirement for attachment. These results indicate that RON4 and RON5 are mainly required for sporozoite attachment and are also involved in the onset of sporozoite movement. The velocity of continuously moving hemolymph sporozoites was reduced approximately 30% by RON4 or RON5 knockdown (Fig. 4B and C), indicating that RON4 and RON5 are dispensable for the motility machinery which allows sporozoites to migrate continuously. As a smaller ratio of RON4-cKD hemolymph sporozoites than RON5-cKD hemolymph sporozoites is able to migrate continuously through the Matrigel, RON4 contribution to sporozoite attachment to the substrate and/or initiation of movement appears to be larger than that of RON5. These results indicate that both RON4 and RON5, as well as RON2 (18), are involved mostly in sporozoite ability to attach to substrates prior to invasion and are not essential for the machinery of motility, in contrast to the crucial roles of the sporozoite micronemal protein TRAP both in attachment and in directional migration (34).

### RON2 and RON4 affect each other for their protein trafficking to rhoptries.

**FIG 5:**
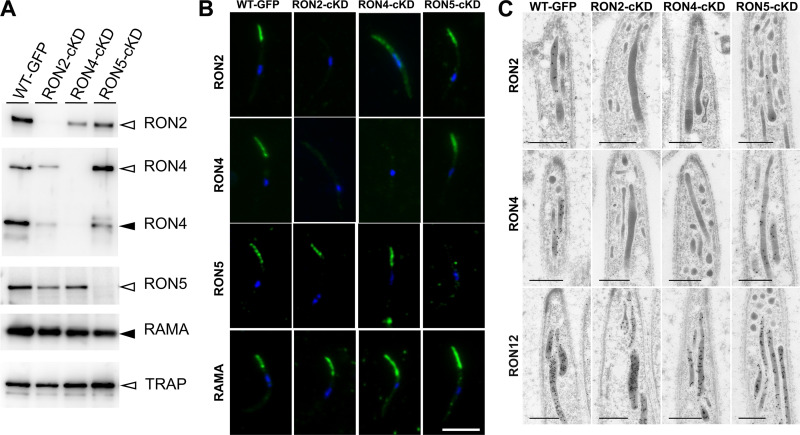
RON2 and RON4 reciprocally affect trafficking to rhoptries in oocyst-derived sporozoites. (A) Western blot analysis of RON complex components in RON2-cKD, RON4-cKD, and RON5-cKD oocyst-derived sporozoites. To investigate the effect of gene knockdowns on rhoptry proteins, homogenates from 100,000 oocyst-derived sporozoites were separated by SDS-PAGE and RON2, RON4, RON5, and RAMA proteins were detected using specific antibodies. TRAP was also detected as a micronemal protein involved in sporozoite motility. The relative intensities of each band were normalized by RAMA and are shown in Fig. S6A. Specific bands corresponding to the full-length and processed forms are indicated by open and closed arrowheads, respectively. (B) Rhoptry neck protein localization analyses in *Pb*WT-GFP, RON2-cKD, RON4-cKD, and RON5-cKD oocyst-derived sporozoites. Sporozoites collected from midguts were fixed by acetone on glass slides and incubated with specific antibodies against RON2, RON4, RON5, or RAMA, followed by incubation with secondary antibodies conjugated to Alexa 488 (green). Nuclei are visualized with DAPI (blue). Bar, 5 μm. Additional images are shown in Fig. S7. (C) Immunoelectron microscopy analyses of RON2, RON4, and RON12 localization in transgenic oocyst-derived sporozoites. RON2 and RON4 localizations to rhoptries were perturbed by RON4 and RON2 knockdown but not by RON5 knockdown. RON12 localization is not affected by repression of any components of the RON complex (lower panels). Bars, 500 nm.

Several rhoptry proteins have been demonstrated to be involved in the trafficking of other proteins to rhoptries. For example, RAMA in P. falciparum merozoites affects the localization of rhoptry-associated protein 1 (RAP1) (28) and RON5 in *Toxoplasma* tachyzoites affects the stabilization of RON2 and RON4 (12). Here, we demonstrated that RON2, RON4, and RON5 interact and that knockdown of each protein in sporozoites resulted in similar phenotypes, namely, the reduction of efficiency in invasion of salivary glands. Therefore, we next investigated rhoptry protein amounts and localization in RON2-cKD, RON4-cKD, and RON5-cKD oocyst-derived sporozoites, to examine whether they function cooperatively in sporozoites. Western blotting analyses using extracts of oocyst-derived sporozoites for each knockdown parasite line confirmed that the amount of targeted protein in these knockdown sporozoites significantly decreased (Fig. 5A and Fig. S6A). The amounts of the processed form of RAMA were not reduced by either RON2, RON4, or RON5 repression, as suggested by immunoelectron microscopy (IEM) analysis (Fig. 2E). In addition, TRAP, a micronemal protein involved in sporozoite motility, is not decreased by knockdown of either RON2, RON4, or RON5. Via RON2 knockdown, the total amount of RON4 is clearly decreased (normalized by the signal density of RAMA) and the RON5 amount is slightly decreased (Fig. S6A). In RON4 knockdown oocyst-derived sporozoites, the RON2 amount is also decreased, while the RON5 amount is not drastically changed (Fig. S6A). In contrast, the effects of RON5 knockdown on other rhoptry neck proteins are limited. However, *ron2* transcription is not affected by either RON4 or RON5 repression, which is demonstrated by real-time RT-PCR using oocyst-derived sporozoites (Fig. S6B).

10.1128/mSphere.00325-20.6 FIG S6 Effect of RON2, RON4, or RON5 knockdown on the expression of other secretory proteins. (A) Quantitative analyses of protein amounts normalized by RAMA amounts in *Pb*WT-GFP, RON2-cKD, RON4-cKD, and RON5-cKD sporozoites (shown in Fig. 5A). The bar graph shows the relative intensities of each band quantified from the Western blotting data shown in Fig. 5A and normalized by those of RAMA. (B) Quantitative analysis of *ron2* mRNA amounts in RON4 or RON5 knockdown sporozoites by real-time RT-PCR. Parasite-infected mosquito midguts were collected at days 15 to 16 postfeeding, and then total RNA was extracted. Real-time RT-PCR was performed three to five times from independently prepared RNA samples. The means for relative *ron2* mRNA amounts compared with *hsp70* mRNA amounts are shown as bars with standard deviation. No significant reduction in *ron2* transcription was detected by either RON4 or RON5 knockdown in sporozoites. Download FIG S6, TIF file, 0.2 MB. Copyright © 2020 Nozaki et al. 2020 Nozaki et al. This content is distributed under the terms of the Creative Commons Attribution 4.0 International license.

10.1128/mSphere.00325-20.7 FIG S7 Effect of RON2 or RON4 knockdown on the localization of rhoptry neck proteins. To support the result shown in Fig. 5B, more IFA images are provided which focus on RON2-cKD and RON4-cKD oocyst-derived sporozoites. Specific antibodies are indicated above the images used to detect RON2, RON4, or RON5. Typical apical-end localizations of RON2 and RON4 were perturbed by RON4 or RON2 knockdown but not by RON5 knockdown. Bars, 10 μm. Download FIG S7, TIF file, 0.5 MB. Copyright © 2020 Nozaki et al. 2020 Nozaki et al. This content is distributed under the terms of the Creative Commons Attribution 4.0 International license.

The localization of RON2, RON4, RON5, and RAMA in oocyst-derived sporozoites was examined by IFA for each knockdown parasite line (Fig. 5B; additional images are shown in Fig. S7). In RON2 knockdown oocyst-derived sporozoites, the RON4 signal intensity is decreased and its typical apical-end localization is disturbed. Likewise, in RON4 knockdown oocyst-derived sporozoites, the apical-end localization of RON2 is perturbed. In contrast, RON5 localizes to the apical end regardless of the knockdown of RON2 or RON4. Moreover, RON5 knockdown does not affect RON2 and RON4 apical-end localization. The precise protein localization in each knockdown sporozoite was determined by IEM. Since the reactivity of anti-RON5 antibodies is insufficient for IEM, antibodies against RON2, RON4, and RON12, another rhoptry neck protein, were used for IEM on oocyst-derived sporozoites (Fig. 5C). RON2 in RON4-cKD oocyst-derived sporozoites and RON4 in RON2-cKD oocyst-derived sporozoites could not be detected by IEM using antibodies at concentrations that detect these proteins in *Pb*WT-GFP sporozoites. It was confirmed that the absence of RON5 did not affect the rhoptry localization of RON2 and RON4.

Taken together, the results indicate that RON2 and RON4 function reciprocally for stability and/or trafficking to rhoptries in sporozoites, whereas RON5 is independent from RON2 and RON4 in terms of its localization to rhoptries.

## DISCUSSION

### RON2, RON4, and RON5 form a complex in sporozoites as well as in merozoites.

To address the question whether invasion mechanisms are conserved between *Plasmodium* infective stages, we determined if the RON complex, composed of RON2, RON4, and RON5, is formed in sporozoites and has roles during the invasion of salivary gland cells. Coimmunoprecipitation assays demonstrated that RON2, RON4, and RON5 interact in protein lysates of oocyst-stage developing sporozoites (Fig. 1B; see also Fig. S2A in the supplemental material). Considering that RON2, RON4, and RON5 localize to rhoptries in sporozoites (16), we conclude that they form a complex in sporozoites in addition to merozoites. RON5 was detected in the RON complex in sporozoites in a smaller amount than in merozoites, raising the possibility that each invasive form may alter the protein components and/or protein ratio of the RON complex to invade different target cells. The RON2 and AMA1 interaction in P. falciparum has been demonstrated to be required for sporozoite infection of hepatoma cells as well as merozoite invasion of erythrocytes, as shown by experiments using the inhibitory peptide R1 (35). Faint signals corresponding to AMA1 were detected in immunoprecipitates using anti-RON4C antibodies and oocyst-derived sporozoite extracts, suggesting that the RON complex may weakly interact with AMA1 (Fig. S2B). Since the AMA1 amount in developing sporozoites might be insufficient for detection using the immunoprecipitation assay, hemolymph sporozoites might be a more suitable material to evaluate the ability of AMA1 to interact with the RON complex and its involvement in salivary gland invasion. However, the number of sporozoites collected from hemocoel preparations is too limited to perform immunoprecipitation assays and thus those assays could not be performed in this study. As it remains controversial whether AMA1 in sporozoites contributes to invasion of salivary glands and/or hepatocytes (14, 27, 35), further studies using biochemical as well as reverse-genetics approaches would be required. Another possibility is that the RON complex has different interaction partners to adjust for invasion of stage-specific target cells. Further investigation would reveal the conserved versus host cell-specific mechanisms of apicomplexan infective-stage parasites to infect target cells.

### RON4 is required for sporozoite invasion of salivary glands.

Sporozoite-stage *ron4* knockdown by promoter exchange resulted in a reduction in the numbers of sporozoites residing in salivary glands, indicating that RON4 is required for sporozoite invasion of salivary glands. Previously, it was demonstrated, using another stage-specific RON4 knockdown system, that RON4 was dispensable for salivary gland invasion but important for hepatocyte infection (27). The discrepancy between the previous report and our results may arise due to the timing of RON4 silencing in sporozoites. The transcription of *ron4* starts in developing sporozoites inside oocysts at day 9 postfeeding, increases during maturation until they are released into the hemocoel, and then decreases after sporozoite invasion of salivary glands (16), although RON4 protein remains in salivary gland-residing sporozoites (Fig. 1A). In the Giovannini et al. paper (27), a thermolabile variant of flippase was used to excise the 3′ untranslated region (UTR) of *ron4*, but its expression was initiated on day 17 postfeeding, when most of the *ron4* mRNA is already present in sporozoites and salivary gland invasion has begun. In contrast, with promoter swapping to the *msp9* promoter, which is active in schizonts but almost silent in sporozoites (18), we successfully repressed *ron4* transcription from the onset of transcription in developing sporozoites. It was confirmed by Western blotting that RON4 remains at an undetectable level in RON4-cKD mature sporozoites (Fig. 2C). Taken together, we conclude that promoter swapping to the *msp9* promoter is a suitable strategy to investigate the roles of rhoptry proteins in sporozoites, including in terms of the invasion of salivary glands.

### RON2, RON4, and RON5 function cooperatively during sporozoite invasion of salivary glands.

We have shown by sporozoite-stage gene knockdowns that RON4 and RON5, in addition to RON2 (18), are involved in sporozoite invasion of salivary glands (Fig. 3). *In vitro* sporozoite motility assays suggest that both RON4 and RON5 are required to adhere to substrates, such as the basal membrane surrounding salivary glands. This is confirmed by *in vivo* confocal images showing that RON4-cKD and RON5-cKD sporozoites did not attach to the surface of salivary glands, demonstrating that they could not adhere to salivary glands prior to invasion. These phenotypes resemble RON2 conditional knockdown sporozoites (18), indicating that RON2, RON4, and RON5 function cooperatively in sporozoites prior to or during invasion of salivary glands. We have reported that RON11, another rhoptry neck protein expressed in both sporozoites and merozoites, is crucial for sporozoite invasion of salivary glands via involvement in attachment and gliding ability (36). RON11 contains seven predicted transmembrane domains and is likely anchored in rhoptries rather than secreted together with the RON complex. Our findings reveal that not only micronemal or surface proteins such as TRAP, TREP/S6/UOS3, and LIMP, but also rhoptry neck proteins, contribute to sporozoite invasion of salivary glands via adhesion to substrates and/or subsequent motility. Further investigation on the functional interactions among rhoptry neck proteins would shed light on these new aspects of sporozoite invasion mechanisms.

Matrigel mimics the environment of sporozoites deposited by mosquitoes into mammalian skin, and this matrix partially compensated for the reduced attachment and gliding ability phenotype of RON5 knockdown sporozoites on glass slides. This indicates that RON5 is mainly involved in sporozoite initial attachment rather than the onset or continuation of gliding. In the case of RON4 knockdown sporozoites, the effect of Matrigel embedding was small, suggesting that RON4 repression has a more drastic impact on sporozoite adhesion or may affect multiple steps such as the initiation of movement.

It is noteworthy that the decreased efficacy of adhesion and salivary gland invasion phenotypes of RON4 knockdown sporozoites is similar to those of RON2 knockdown sporozoites. This is possibly because RON2 or RON4 knockdowns reciprocally perturb localization to rhoptries (Fig. 5C), resulting in the functional disruption of both RON2 and RON4 in sporozoites. This could be the reason that RON2-cKD and RON4-cKD sporozoites demonstrate more severe phenotypic changes than RON5-cKD sporozoites, in which RON2 and RON4 normally localize to rhoptries.

### RON2 and RON4 affect each other to stabilize proteins and/or trafficking to rhoptries.

Many studies have been conducted using the extracellular administration of inhibitory antibodies or peptides to demonstrate the importance of RON2 and AMA1 interaction during the invasion of target cells by *Plasmodium* and *Toxoplasma* infective stages (9, 37, 38). However, due to the inability to obtain transgenic parasites harboring disrupted genes of rhoptry neck proteins, only a few studies have addressed their functions prior to secretion. For example, *ron5* knockdown in *Toxoplasma* tachyzoites resulted in RON2 degradation and mistargeting of RON4, resulting in failure to invade host cells (12). Here, by repressing *ron2* or *ron4* expression specifically in sporozoites, we demonstrated that both RON2 and RON4 are reduced in quantity and are not localized to rhoptries (Fig. 5). Taking into consideration that *ron2* mRNA amounts are not affected by RON4 knockdown (Fig. S6B), they probably have functions to mutually stabilize and/or transport each other via interaction. Notably, this effect by RON2 or RON4 repression is neither through rhoptry formation nor through basic protein trafficking to organelles, as RAMA and RON12 are correctly localized to normal-appearing rhoptries (Fig. 2E and Fig. 5C). Furthermore, in contrast to *Toxoplasma* tachyzoites, in *Plasmodium* sporozoites the contributions of RON5 to RON2 and RON4 trafficking to rhoptries and vice versa are not observed (Fig. 5C). This could be explained by the fact that the primary sequence of the RON2-interacting region of Toxoplasma gondii RON5 (*Tg*RON5) (amino acids 1292 to 1775) is not highly conserved in *Plasmodium* RON5. In addition, *Tg*RON5 is detected as processed, although the processing is not essential for its function, while *Plasmodium* RON5 is detected as full-length protein in both sporozoites and schizonts. However, the overall mechanisms to stabilize or to transport rhoptry neck proteins via their interaction might be well conserved across the Apicomplexa phylum.

In this study, two possibilities regarding RON4 functions in sporozoite invasion could not be distinguished: (i) if RON4, as well as RON2, has direct function for adhesion versus (ii) if RON4 solely contributes to RON2 stability and trafficking to rhoptries, which is then crucial for invasion. The latter possibility is less likely, because the RON4-cKD phenotype is slightly more severe than the RON2-cKD phenotype with respect to the efficiency of salivary gland invasion (18). For further elucidation of the mechanisms, the regions involved in direct protein interaction need to be determined for *Plasmodium* RON2, RON4, and RON5. Then, it might be possible to evaluate the importance of complex formation during sporozoite invasion by generating transgenic parasites expressing mutant RON2, RON4, or RON5 proteins which cannot interact with each other.

## MATERIALS AND METHODS

### Experimental animals.

Female ICR mice and Wistar rats were purchased from CLEA Japan (Tokyo, Japan) and maintained in our animal facility. All mice were 4 to 8 weeks old at the time of blood-stage parasite infection. Animal experimental protocols were approved by the Institutional Animal Care and Use Committee, Ehime University, Japan. All experiments were conducted according to the Ethical Guidelines for Animal Experiments of Ehime University.

### Parasites and mosquitoes.

Anopheles stephensi mosquitoes (SDA 500 strain) were reared using standard protocols (39) and maintained on a 5% sucrose solution as adults. All parasites were derived from a P. berghei ANKA strain which expresses GFP under the control of the *elongation factor 1A* (*ef1α*) promoter without a drug-resistant cassette (*Pb*WT-GFP) (40), kindly provided by C. J. Janse, Leiden University, Netherlands. Transgenic parasites expressing C-terminal c-Myc-tagged RON2, RON4, or RON5 were generated previously (16). Cryopreserved P. berghei-infected erythrocytes were inoculated into 4- to 6-week old female ICR mice (CLEA Japan) via intraperitoneal injection to obtain asexual-stage parasites. Approximately 60,000 parasitized erythrocytes were transferred intravenously into a naive mouse 5 days before mosquito feeding. When the parasitemia reached 5 to 10% and the number of exflagellation centers had reached 30 per 1 × 10^5^ erythrocytes, the infected mice were fed on by a group of female mosquitoes. Fully engorged mosquitoes were selected and maintained at 20°C until use.

### Production of recombinant proteins and preparation of anti-PbRON4 and anti-PbRON5 antibodies.

To generate RON4 polyclonal antibodies, a partial recombinant protein corresponding to the C-terminal region (amino acids 565 to 786) of RON4 (PBANKA_0932000, RON4C), which is highly conserved in amino acid sequence among *Plasmodium* species, was produced using the wheat germ cell-free protein synthesis system (CellFree Sciences, Matsuyama, Japan) (41). Briefly, DNA encoding *Pb*RON4 was amplified by PCR from genomic DNA of *Pb*WT-GFP using specific primers tailed with EcoRV and BamHI restriction enzyme recognition sites (shown in Table S1 in the supplemental material). The DNA fragment was inserted into the pEU-E01-GST-TEV-MCS vector (CellFree Sciences) to produce GST-tagged RON4 at its N terminus. After transcription and translation using the wheat germ cell-free protein expression system, GST-tagged RON4 protein was purified using a glutathione-Sepharose 4B column (GE Healthcare UK, Buckinghamshire, United Kingdom). For production of polyclonal antibodies recognizing RON5 protein (PBANKA_0713100), the C-terminal region (amino acids 861 to 1148) was produced as described above.

10.1128/mSphere.00325-20.8 TABLE S1 Primers and sequences used in the study. Download Table S1, DOCX file, 0.02 MB. Copyright © 2020 Nozaki et al. 2020 Nozaki et al. This content is distributed under the terms of the Creative Commons Attribution 4.0 International license.

Japanese white rabbits were immunized subcutaneously three times with 250 μg of purified recombinant protein with Freund’s adjuvant, and antisera were obtained 14 days after the final immunization (Kitayama Labes, Ina, Japan). The reactivity and specificity of anti-RON4 and anti-RON5 antisera were determined by Western blotting. Specific antibodies against RON4 or RON5 were affinity purified using recombinant proteins covalently conjugated to HiTrap *N*-hydroxysuccinimide (NHS)-activated high-performance (HP) columns (GE Healthcare).

### Western blotting.

Protein extracts from schizonts or sporozoites or following coimmunoprecipitation were preincubated in SDS sample buffer containing 10% 2-mercaptoethanol at 95°C for 5 min (for RON4 and RON5 detection) or 4°C overnight (for RON2 detection) (18), separated on 5 to 20% gradient acrylamide gels (ATTO, Tokyo, Japan), and transferred by electroblotting to polyvinylidene difluoride (PVDF) membranes (Millipore, Burlington, VT, USA). Membranes were blocked with Blocking One buffer (Nacalai Tesque, Kyoto, Japan) and incubated with specific antibodies against RON2, RON4, RON5, or rhoptry-associated membrane antigen (RAMA, PBANKA_0804500) diluted with phosphate-buffered saline (PBS) containing 0.1% Tween 20 (1:2,500, 1:500, 1:500, or 1:2,500, respectively) for 1 h at room temperature followed by incubation with anti-rabbit IgG antibodies conjugated with horseradish peroxidase (1:25,000) at 37°C for 45 min (18). Anti-c-Myc rabbit monoclonal antibodies (71D10, Cell Signaling Technology, Danvers, MA, USA) were used to detect RON4-c-Myc or RON5-c-Myc proteins at 1:500 dilution. To detect the micronemal proteins AMA1 (PBANKA_0915000) and TRAP (PBANKA_1349800), anti-AMA1 and anti-TRAP antibodies were used (36, 42). Secondary antibody signals were developed using the Immobilon Western chemiluminescent horseradish peroxidase (HRP) substrate (Millipore) and detected using the ImageQuant LAS4000 imaging system (GE Healthcare). The list of antibodies used in this study is shown in Table S2.

10.1128/mSphere.00325-20.9 TABLE S2 List of antibodies used in the study. Download Table S2, DOCX file, 0.02 MB. Copyright © 2020 Nozaki et al. 2020 Nozaki et al. This content is distributed under the terms of the Creative Commons Attribution 4.0 International license.

### Coimmunoprecipitation assay.

For schizont preparations, *Pb*WT-GFP-infected Wistar rat blood was collected and cultured in 20% fetal calf serum (FCS) containing RPMI 1640 medium for 16 h at 36.5°C and schizont-infected erythrocytes were purified using Nycoprep 1.077 solution (Axis-Shield, Dundee, United Kingdom) (43). After treatment with 0.08% saponin for 15 min on ice, schizont pellets were stored at −80°C until use. Sporozoites were collected from A. stephensi midguts 19 to 21 days postfeeding. The midguts were ground in PBS containing protease inhibitor cocktail set III (Millipore) and 0.5 mM EDTA and purified by centrifugation at 2,500 × *g* at room temperature for 20 min on a 17% Accudenz cushion (Accurate Chemical, Westbury, NY, USA) (44). Sporozoite pellets were stored at −80°C.

Schizont and sporozoite pellets were resuspended in lysis buffer (1% CHAPS, protease inhibitor cocktail set III [Millipore], 0.5 mM EDTA, 1 mM phenylmethylsulfonyl fluoride [PMSF], 0.15 mM aprotinin in PBS) and sonicated using a Covaris focused-ultrasonicator S220 (Covaris Inc., Woburn, MA, USA; settings, 5% duty cycle, intensity 105, cycle per burst 200, time 15 s). After centrifugation at 19,500 × *g* at 4°C for 5 min, the supernatants were preabsorbed with protein G-Sepharose 4 beads (GE Healthcare). Recovered supernatants were incubated with antibodies (anti-RON4, anti-RON2, or anti-GST) with gentle rotation at 4°C for 2 h, and then protein G-Sepharose 4 beads (GE Healthcare) were added to capture antibody-bound proteins. After 1 h of incubation at 4°C, the beads were washed with sequential single washes of NETC (50 mM Tris-HCl, 0.15 M NaCl, 1 mM EDTA, and 0.5% CHAPS) with 0.5% bovine serum albumin (BSA), NETC, high-salt NETC (0.5 M NaCl), NETC, and low-salt NETC (0.05 M NaCl and 0.17% CHAPS). The immunoprecipitated proteins were extracted by incubation with sample buffer solution for SDS-PAGE (Nacalai Tesque) containing 10% 2-mercaptoethanol at 4°C overnight followed by Western blotting analyses.

### Transgenic parasite generation.

To repress *ron4* or *ron5* transcription only in sporozoites, their native promoter regions were replaced by homologous recombination with the promoter region of a merozoite-specific expressing gene (*merozoite surface protein 9* [*msp9*]) (see Fig. S3A and B) (18). DNA fragments for the homologous recombination cassettes (RON4-N, −17 to +869 bp from the start codon; *ron4*-upstream, −1047 to −459 bp; RON5-N, −65 to 602 bp; and *ron5*-upstream, −1344 to −595 bp) were amplified from genomic DNA of *Pb*WT-GFP by PCR and ligated to the transgenic vectors containing an *msp9* promoter region and a human dihydrofolate reductase (hDHFR)-expressing cassette to confer antimalarial drug resistance. The *msp9* promoter regions in transgenic vectors were replaced by the *ron4* and *rap1* native promoters to generate RON4 control (RON4-cont) and RON5 control (RON5-cont) parasites, respectively.

The transgenic vectors were linearized with XhoI and transfected into schizont-enriched *Pb*WT-GFP parasites by electroporation using Nucleofector (Lonza, Basel, Switzerland) (43). Transgenic parasites were selected by adding 70 μg/ml of pyrimethamine to the drinking water after parasite inoculation into 4-week-old ICR female mice. Transgenic parasites were cloned by limiting dilution. The sequences of all primers used in this study are listed in Table S1.

### Genomic Southern blot analysis.

Genotypes of the cloned parasites were confirmed by Southern blotting. Genomic DNA was isolated from *Pb*WT-GFP and cloned transgenic parasite-infected erythrocytes using a Wizard Genomic DNA purification kit (Promega, Madison, WI, USA) and digested with the restriction enzymes BamHI and EcoRV overnight. The DNA fragments were electrophoresed on 0.8% agarose gels and transferred to a Hybond-N+ nylon membrane (GE Healthcare) using the alkaline transfer method. A DNA fragment corresponding to hDHFR was amplified with PCR and labeled by an AlkPhos direct labeling kit (GE Healthcare). The probes were hybridized onto membranes at 55°C, and chemiluminescent signals were developed and detected using a CDP-Star reagent and an image analyzer (ImageQuant LAS 4000; GE Healthcare) (36).

### Real-time RT-PCR.

To confirm the reduction of *ron4* or *ron5* transcript levels in RON4-cKD or RON5-cKD sporozoites, real-time reverse transcription (RT)-PCR was carried out using specific primers as shown in Table S1. Total RNA was extracted from 10 to 15 infected mosquito midguts at days 14 to 15 postfeeding using the RNeasy Micro kit (Qiagen GmbH, Hilden, Germany). After DNase treatment, cDNA was synthesized using a PrimeScript RT reagent kit (Perfect Real Time; TaKaRa Bio, Otsu, Japan). Quantitative RT-PCR was performed using the TaKaRa PCR Thermal Cycler Dice with a SYBR Premix *Ex Taq* (TaKaRa Bio) and specific primers. A primer set for detection of *heat shock protein-70* (*hsp70*, PBANKA_0711900) was used for normalization (36). Analysis of transcript levels relative to the average level of the internal control gene was calculated using the delta-*C_T_* method (45). The experiment was performed at least four times using independently prepared samples from different infections, and the mean and standard deviations were determined.

### Comparison of the numbers of sporozoites collected from mosquito bodies.

Oocyst numbers of at least five mosquito midguts were counted at day 12 postfeeding to select mosquito groups with >60% prevalence for further experiments. Sporozoites were collected and counted from midguts, hemolymph, and salivary glands of 20 to 30 infected A. stephensi mosquitoes at days 24 to 26 postfeeding. Experiments were repeated more than four times. The difference in the sporozoite numbers among parasite lines was analyzed by the Kruskal-Wallis test with a Dunn *post hoc* test.

### Sporozoite gliding assay.

At days 17 to 22 postfeeding, sporozoites were collected from hemolymph by RPMI 1640 medium infusion through the mosquito thorax. For gliding assays on glass slides, sporozoites were mixed with the same volume of RPMI 1640 medium containing 20% fetal calf serum (FCS) and placed in a glass-bottom dish. For gliding assays in Matrigel, sporozoites in RPMI 1640 containing 20% FCS were mixed with an equal volume of Matrigel (Corning, Corning, NY, USA), placed on glass slides, and covered with coverslips. Sporozoite movement was detected by GFP fluorescence using an Axiovert inverted fluorescence microscope (Carl Zeiss, Oberkochen, Germany) and recorded with an AxioCam MRm charge-coupled device camera (Carl Zeiss) every 2 s for up to 150 frames (5 min). Sporozoites on glass slides were classified manually as gliding, waving, or drifting according to the work of Hegge et al. (46). Experiments were repeated five times for each parasite line. The effect of RON2, RON4, or RON5 repression on sporozoite motility was evaluated by comparison with the motile patterns of *Pb*WT-GFP sporozoites. Statistical analysis was performed using the one-way analysis of variance (ANOVA) test with the Tukey multiple-comparison test. In Matrigel, sporozoites were categorized as moving in a circular mode (circulating), meandering, or nonmotile, according to the work of Volkmann et al. (33). Experiments were repeated four times with at least 200 sporozoites for each line. The velocity of gliding sporozoites was calculated using the MTrack2 plugin in Fiji software (NIH, Bethesda, MD). Statistical analysis was performed using the Mann-Whitney *U* test.

### Immunofluorescence analysis (IFA).

Sporozoites isolated from midguts or hemolymph of infected mosquitoes were spotted on glass slides, immediately air-dried, and fixed with cold acetone for 3 min. Slides were blocked with PBS containing 10% FCS for 30 min at 37°C and incubated with primary antibodies diluted in PBS containing 10% FCS for 2 h at 37°C. After washing with PBS, slides were incubated with fluorescence-conjugated secondary antibodies (Alexa Fluor 488 goat anti-rabbit IgG, 1:500; Thermo Fisher Scientific, Waltham, MA, USA) for 45 min at 37°C. The samples were mounted in ProLong Gold antifade reagent (Thermo Fisher Scientific) and observed under a fluorescence microscope (Axio Observer z1; Carl Zeiss). Differential interference contrast (DIC) and fluorescence images were obtained using a charge-coupled device camera (AxioCam MR; Carl Zeiss). Images were processed using Image J software (47).

### Immunoelectron microscopy (IEM).

Midguts of mosquitoes infected with *Pb*WT-GFP, RON2-cKD, RON4-cKD, and RON5-cKD were collected by dissection on day 16 postfeeding, fixed in 1% paraformaldehyde-0.2% glutaraldehyde, and embedded in LR-White resin (Polysciences, Warrington, PA, USA) (18). Ultrathin sections were blocked in PBS containing 5% nonfat milk and 0.01% Tween 20 (PBS-MT) and then incubated at 4°C overnight with specific antibodies against RON2, RON4, RON12 (PBANKA_0501400), or RAMA (48). The sections were washed with PBS containing 0.4% Block Ace powder (DS Pharma, Osaka, Japan) and 0.01% Tween 20 (PBS-BT), and the grids were incubated for 1 h at 37°C with goat anti-rabbit IgG conjugated with 15-nm gold particles (GE Healthcare) diluted 1:20 in PBS-MT and were rinsed with PBS-BT. The sections were then stained with uranyl acetate and lead citrate. Samples were examined using a transmission electron microscope (JEM-1230; JEOL, Akishima, Japan).
