# Supplementary figures and images for: Detection of the Rhoptry Neck Protein Complex in Plasmodium Sporozoites and Its Contribution to Sporozoite Invasion of Salivary Glands
Source: mSphere. 2020 Aug 19;5(4):e00325-20. doi: 10.1128/mSphere.00325-20 (PMC7440843; doi:10.1128/mSphere.00325-20)

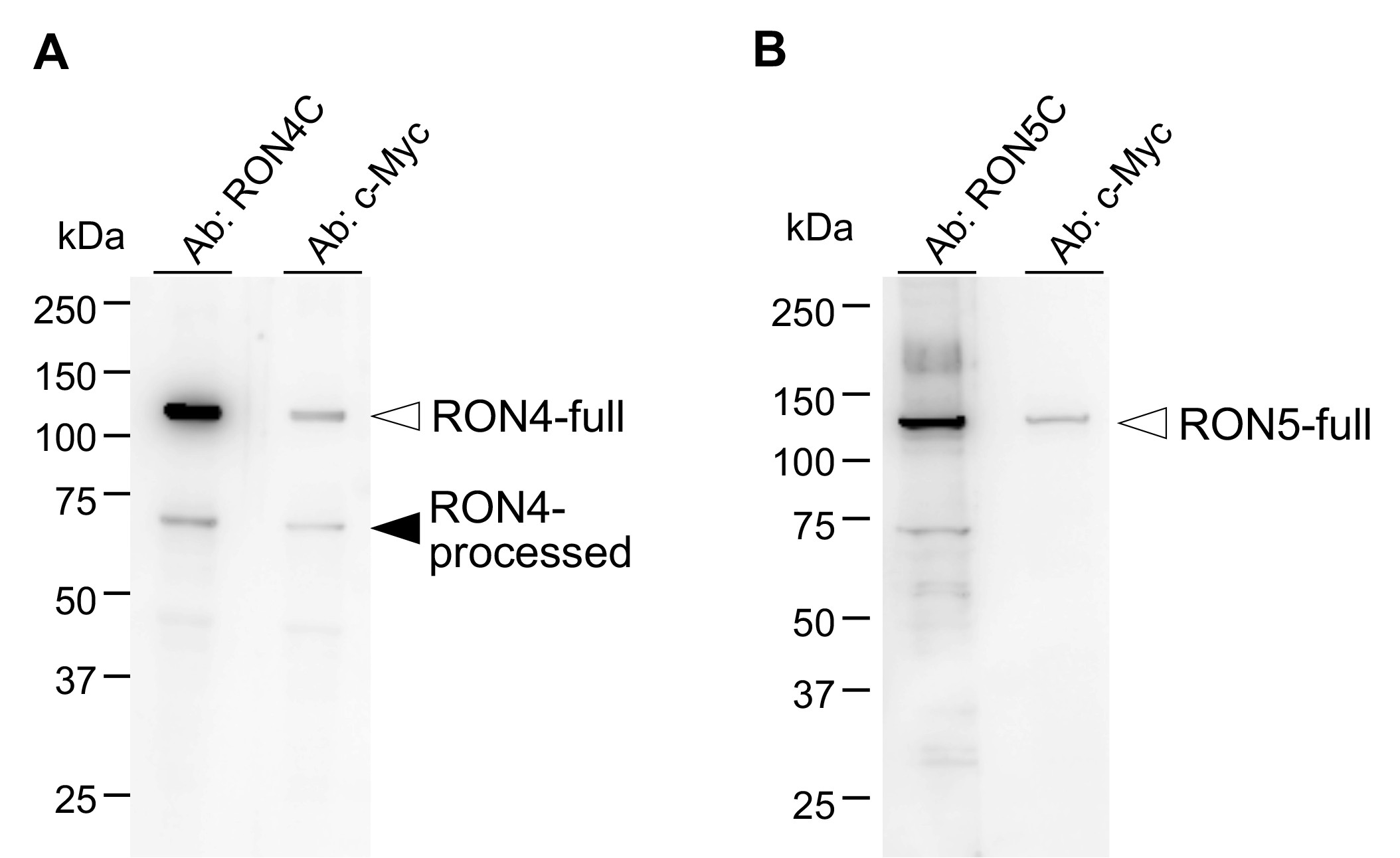

Supplement: FIG S1 [file mSphere.00325-20-sf001.tif]

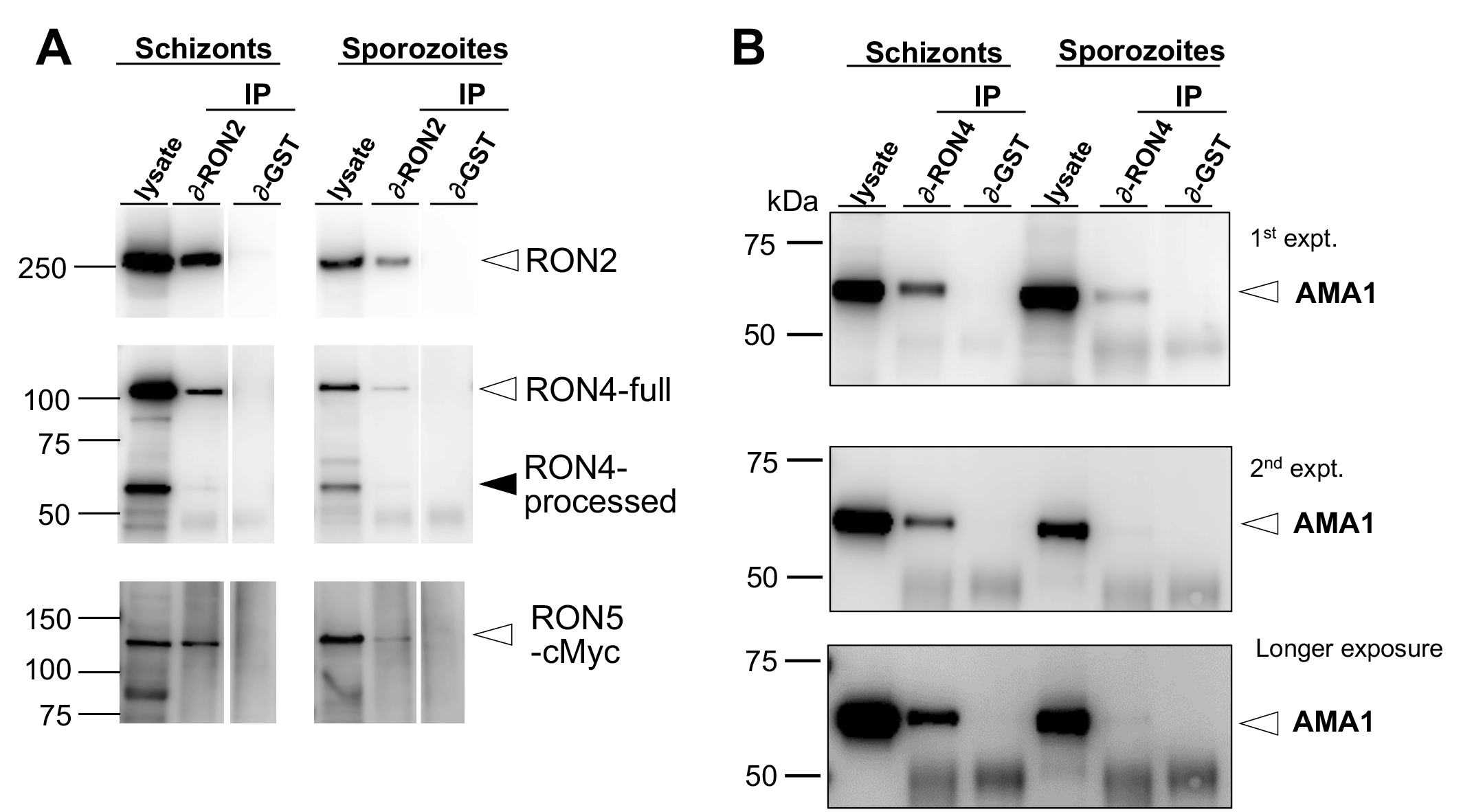

Supplement: FIG S2 [file mSphere.00325-20-sf002.tif]

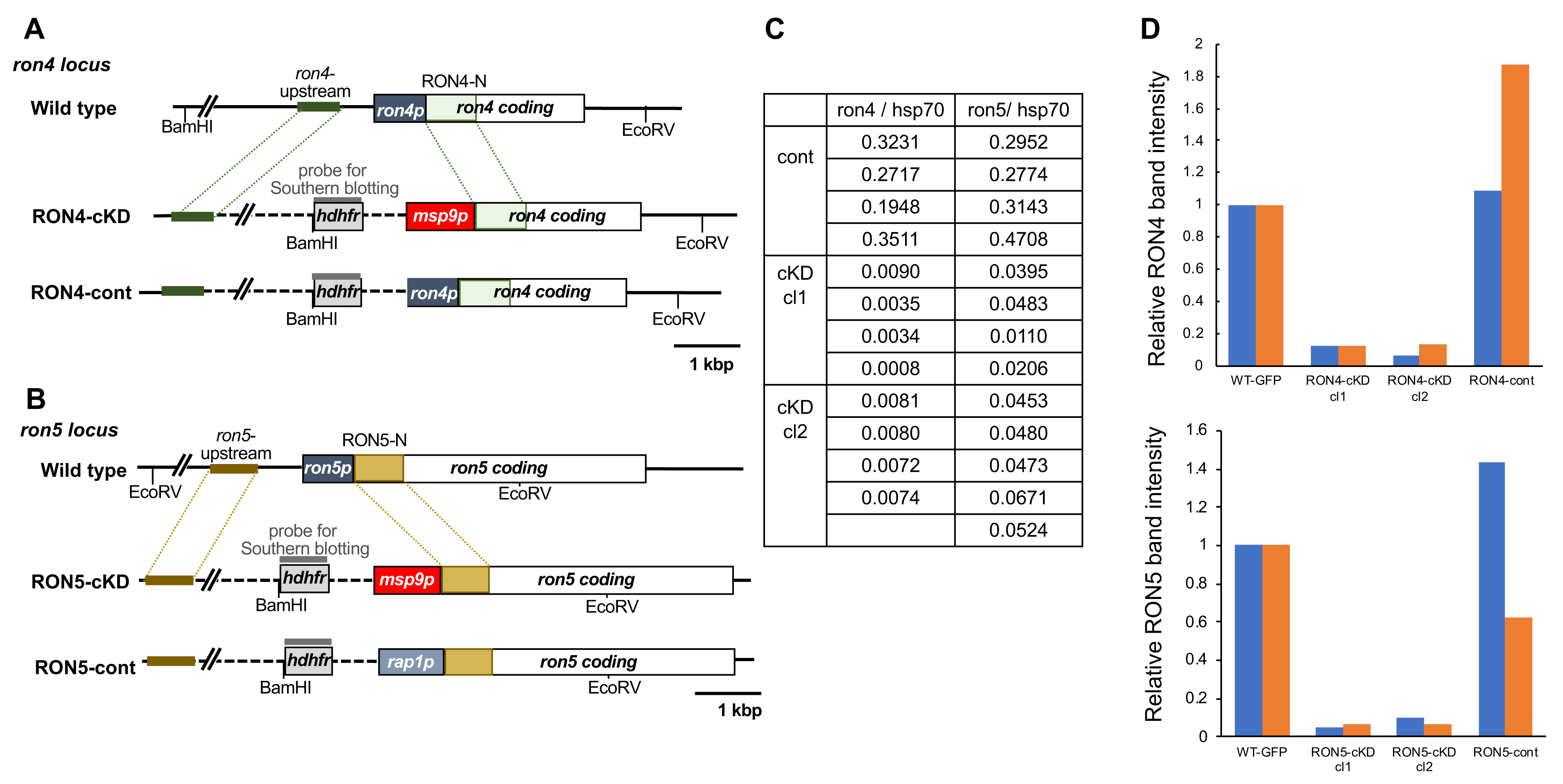

Supplement: FIG S3 [file mSphere.00325-20-sf003.tif]

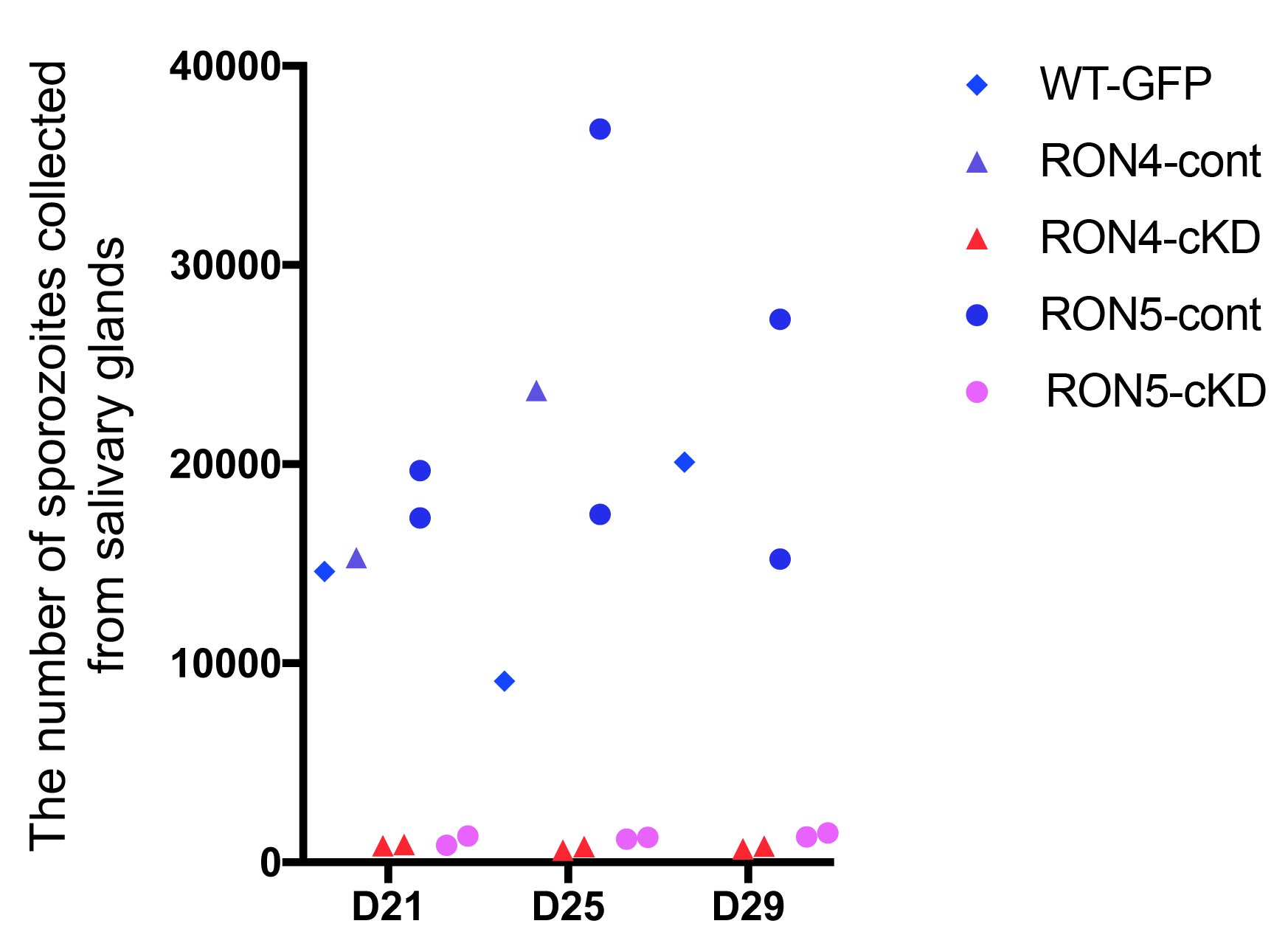

Supplement: FIG S4 [file mSphere.00325-20-sf004.tif]

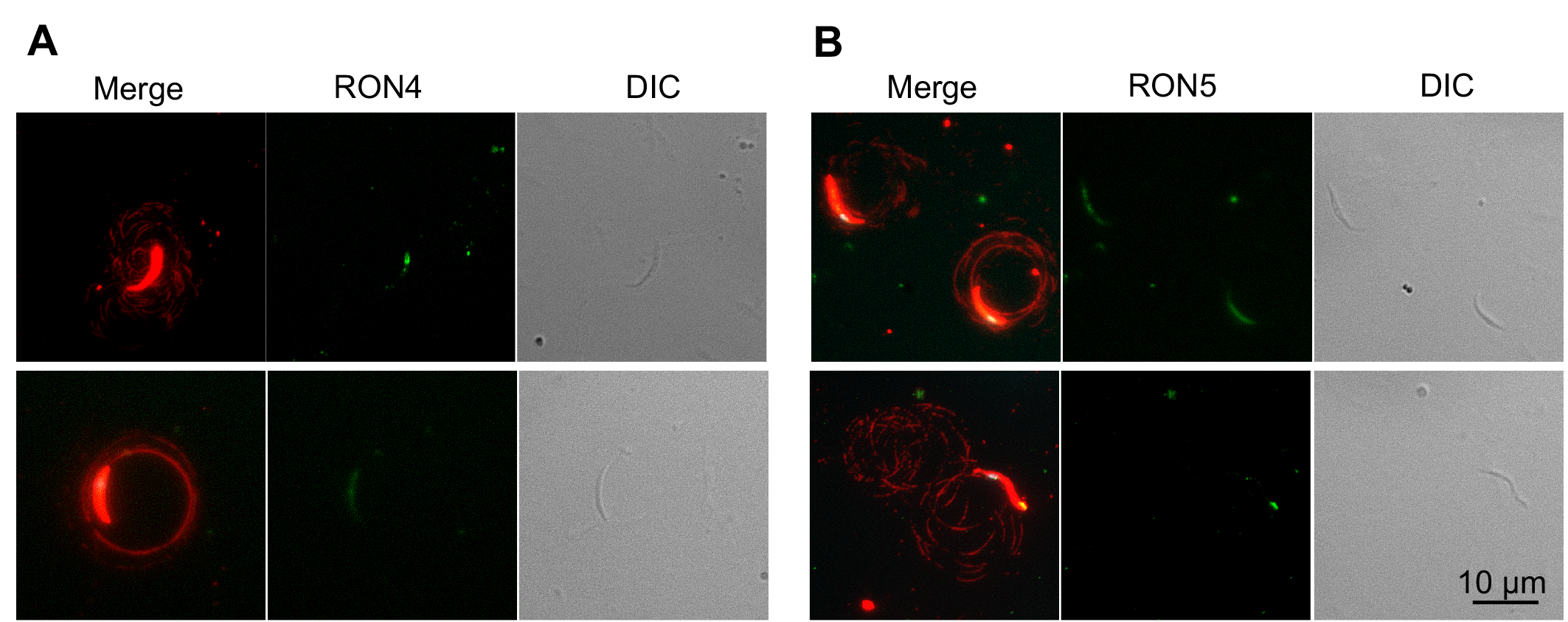

Supplement: FIG S5 [file mSphere.00325-20-sf005.tif]

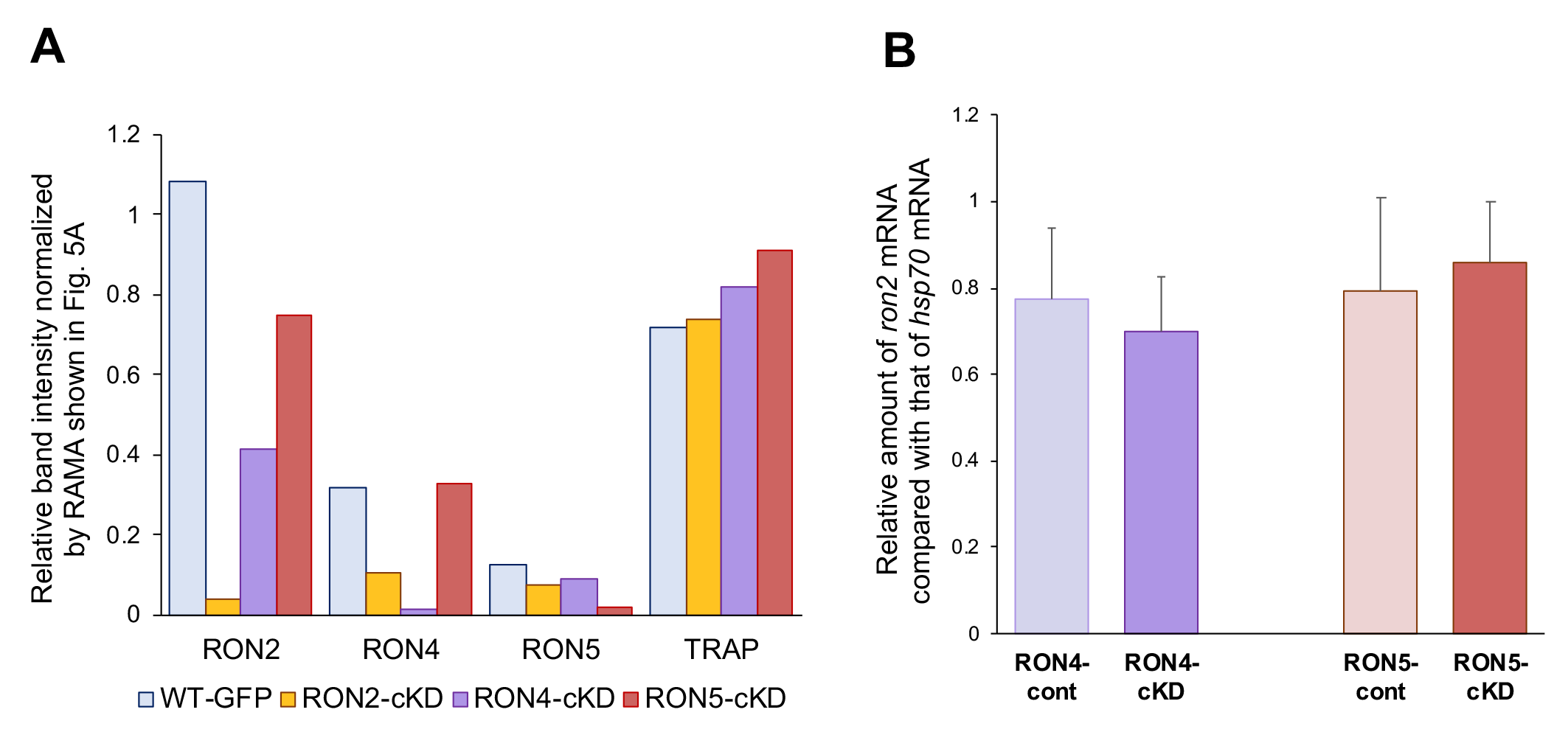

Supplement: FIG S6 [file mSphere.00325-20-sf006.tif]

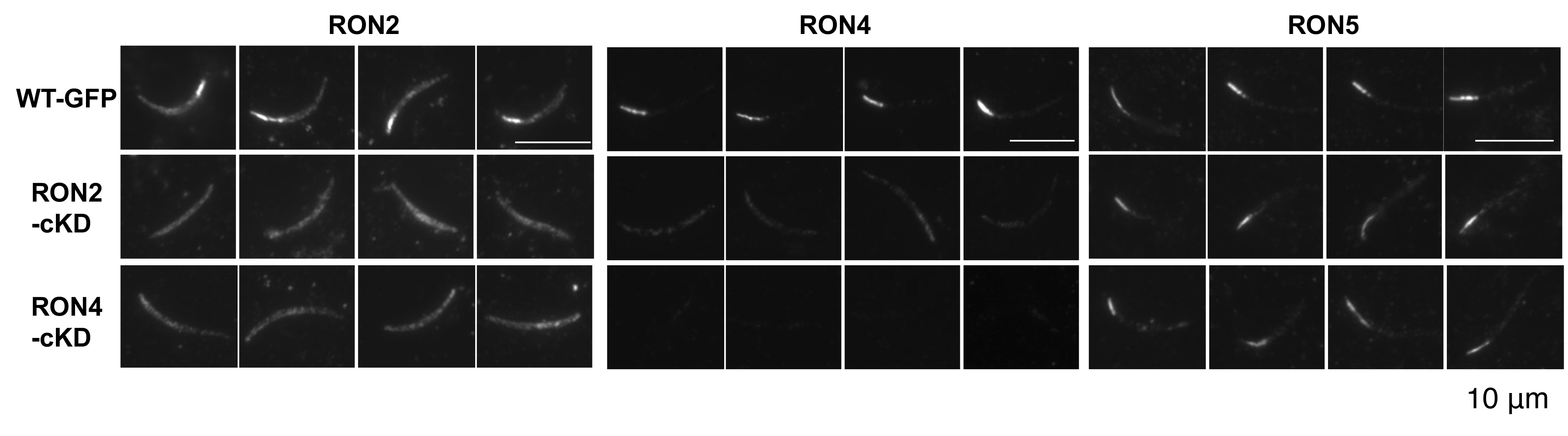

Supplement: FIG S7 [file mSphere.00325-20-sf007.tif]
