# Supplementary material for: Detection of the Rhoptry Neck Protein Complex in Plasmodium Sporozoites and Its Contribution to Sporozoite Invasion of Salivary Glands
Source: mSphere. 2020 Aug 19;5(4):e00325-20. doi: 10.1128/mSphere.00325-20 (PMC7440843; doi:10.1128/mSphere.00325-20)
Supplement: TABLE S1 [file mSphere.00325-20-st001.docx]

**Table S1. Lists of primers used in this study.**

| **Primer** | **Sequence (5‘--->3’)** |
| --- | --- |
| **Transgenic parasite construction** | |
| RON4-cKD-N-F BglII | GAAGATCTAAAATATATAATAAAATGTCTGGCC |
| RON4-cKD-N-R XhoI | CCGCTCGAGACTGTTGTGTGCATATATATCAC |
| RON4-cKD-upstream-F XhoI | CCGCTCGAGATACACGGGTATGCATGCGTATGAA |
| RON4-cKD-upstream-R KpnI | GGGGTACCGCATTTTTTGGTAGCAGTATATCAATG |
| RON4-cont F-ApaI | CCgggcccGCCTACACCGTGTTTAGCTTCTAC |
| RON4-cont R-XhoI | CCGctcgagCAAGTATGAAAACGTTTCATTCGT |
| RON5-cKD-N-F BglII | GAAGATCTGTGCGATTTGATTTAATTGTTCTTT |
| RON5-cKD-N-R XhoI | CCGCTCGAGCTTTCTAGTTTCGTTGCCATGTT |
| RON5-cKD-upstream-F XhoI | CCGCTCGAGATTATTGCTGTGAAGAAAATGATATTTA |
| RON5-cKD-upstream-R KpnI | GGGGTACCGGATAAGGAAAATGTGAAAATACAC |
| msp9_promoterF_ApaI | AGGGGGCCCTCTATGAAATTAAAAGAGTAAACC |
| msp9_promoterR_BglII | GAAGATCTTTCTTAATAAAGAATAGTAATTTGTTT |
| rap1_promotorF_ApaI | AGGGGGCCCATGCAATTCACAGCATATTAAAATCTA |
| rap1_promotorR_BglII | GGAAGATCTTTTTCGCAAAAAAAATAAAATAATAATAAAAT |
| **Southern blot probe** | |
| hDHFR southern F: | GGTTCGCTAAACTGCATCGT |
| hDHFR southern R: | ATTGCCTTTCTCCTCCTGGAC |
| **Real time PCR** | |
| RON2 RT-F | CGTCTACATCGGCCTTTATTC |
| RON2 RT-R | GCGATAGCATGTGTTGTAAATTGG |
| HSP70_RT-F: | GGTGATACCCATTTAGGTGGTG |
| HSP70_RT-R: | TTGCTCTTTCGCATTGTGTTC |
| RON4 RT-F | GCTACTTATTAGAGAGCGAAAAC |
| RON4 RT-R | CATGTGCTAATACGTTGTGTG |
| RON5 RT-F | ATGCAAGGAACTGACAAGCA |
| RON5 RT-R | ACAACACTGTCTGGCCTCAC |
| **Recombinant protein for antibody production** | |
| PbRON4C-F-EcoRV | ATCGATATCGAAGAACATAGTAAATCTTTAGAAC |
| PbRON4C-R-BamHI | CGGGATCCTCAACCGTATTCATCAGTAGATAGCCA |
| PbRON5C-F-XhoI | CCGCTCGAGATGCAAGGAACTGACAAGCATATC |
| PbRON5C-R-SpeI | GGACTAGTTAAGGTATTCTTGTATGAACAATAAT |
