# Supplementary material for: Detection of the Rhoptry Neck Protein Complex in Plasmodium Sporozoites and Its Contribution to Sporozoite Invasion of Salivary Glands
Source: mSphere. 2020 Aug 19;5(4):e00325-20. doi: 10.1128/mSphere.00325-20 (PMC7440843; doi:10.1128/mSphere.00325-20)
Supplement: TABLE S2 [file mSphere.00325-20-st002.docx]

**Table S2. Lists of antibodies used in this study.**

| Protein | Accession No. | Amino acid | Protein production | Reference |
| --- | --- | --- | --- | --- |
| RON2 | PBANKA_1315700 | 22–91 | WGCS | Ishino et al, 2019 |
| RON4 | PBANKA_0932000 | 565-786 | WGCS |  |
| RON5 | PBANKA_0713100 | 861-1148 | WGCS |  |
| RAMA | PBANKA_0804500 | 251-635 | WGCS | Ishino et al, 2019, Tokunaga et al, 2019 |
| TRAP | PBANKA_1349800 | 26–541 | WGCS | Ishino et al, 2019, Bantuchai et al, 2019 |
| AMA1 | PYYM_091600 | 22-479 | WGCS | Ishino et al, 2019 |
| RON12 | PY00202 | 26–255 | WGCS | Oda-Yokouchi et al., 2019 |
